# Supplementary material for: Host RNA Expression Signatures in Young Infants with Urinary Tract Infection: A Prospective Study
Source: Int J Mol Sci. 2024 Apr 29;25(9):4857. doi: 10.3390/ijms25094857 (PMC11084417; doi:10.3390/ijms25094857)
Supplement: Supplementary file 1 [file ijms-25-04857-s001.zip › ijms-2936987-supplementary.pdf]

Supplemental Material

Table S1. Pathogens in the young infants with UTI with bacteremia, UTI without bacteremia, and definite viral infection.

**UTI with bacteremia** (n=7)	**UTI without bacteremia** (n=46)	**Definite viral infection** (n=33)
Escherichia coli (n=6)	*Escherichia coli* (n=41)	Rhinovirus (n=12)
Enterobacter species (n=1)	*Enterococcus species* (n=4)	Respiratory syncytial virus (n=9)
	Enterobacter species (n=1)	Parainfluenzavirus (n=3)
		Enterovirus (n=2)
		Sars-CoV-2 (n=2)
		Coronavirus OC43 (n=1)
		Influenza A virus (n=1)
		Rotavirus (n=1)
		Varicella zoster virus (n=1)

A

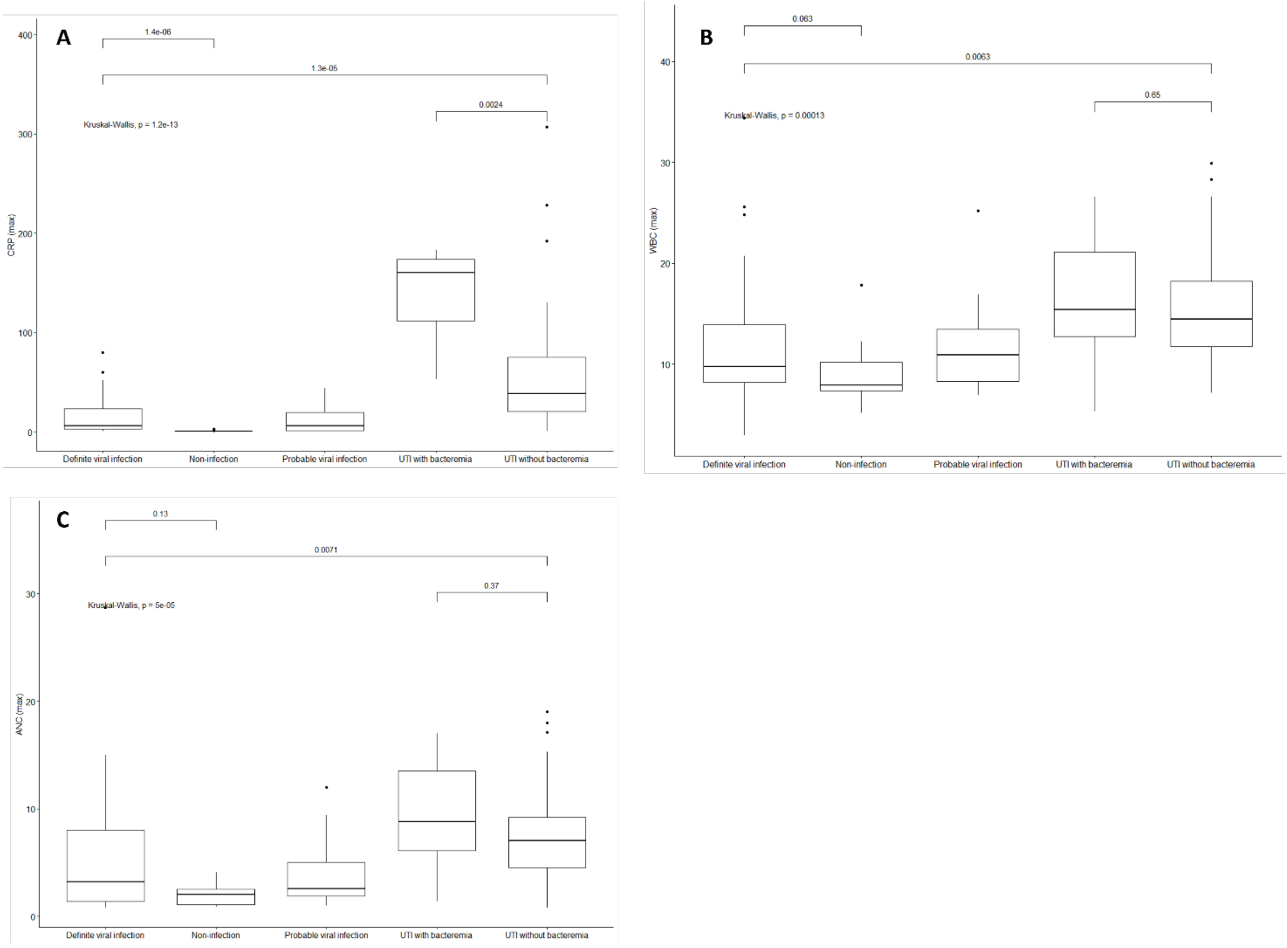

Figure S1. Pairwise comparisons of **A**) C-reactive protein (max), **B**) white blood cell count (max) and **C**) absolute neutrophilic count (max) among groups of young infants with UTI with bacteremia, UTI without bacteremia, definite and probable viral infection and non-infection. Statistics: Kruskal Wallis test and Dunn's post-hoc analysis.

Table S2. The top upregulated genes in young infants with UTI without bacteremia compared to definite viral infection.

Symbol	Log2FC	P-adj	Function (source: www.genecards.com)
RP11-429G19.3	1.12	<0.0001	No annotation.
RP11-356C4.6	1.88	<0.0001	No annotation.
IL10RB-AS1	0.66	<0.0001	RNA gene affiliated with the lncRNA class.
CLRN1	1.99	<0.0001	Development and homeostasis inner ear and retina.
FAM20A	1.03	<0.0001	Likely secreted and may function in hematopoiesis.
AC013461.1	0.89	<0.0001	No annotation.
TRPS1	0.62	<0.0001	Transcription factor that represses GATA-regulated genes.
CD44	0.60	<0.0001	Cell-cell interactions, cell adhesion and migration. Activation, recirculation, and homing of T-lymphocytes. Hematopoiesis, inflammation, and response to bacterial infection.
LTA4H	0.90	<0.0001	Synthesis of leukotriene B4, a proinflammatory mediator. Degrading proline-glycine-proline (PGP), a neutrophil chemoattractant.
ANKRD50	0.61	<0.0001	Endocytic recycling.
SETD7	0.51	<0.0001	Histone-lysine N-methyltransferase activity and p53 binding activity. In response to DNA damage stimulus and heterochromatin organization.
KIAA1324	1.64	<0.0001	Apoptosis and cell proliferation.
TMCO3	0.66	<0.0001	Export of monovalent cations and import of protons across cellular membranes.
RP11-67L2.2	0.59	<0.0001	No annotation.
HSPBAP1	0.45	<0.0001	Binds to one of the small heat shock proteins, specifically hsp27.
SUMF1	0.38	<0.0001	Enzyme that catalyzes the hydrolysis of sulfate esters.

Gene annotations were obtained from www.genecards.com.

Table S3. The top differentially expressed genes (n=50) in young infants with UTI without bacteremia compared to definite viral infections.

no	symbol	alias	log2FC	p-adj	function (source: www.genecards.com)
1	AXL	AXL Receptor Tyrosine Kinase	-1.95	2.05e-11	Receptor tyrosine kinase, cell survival, cell proliferation, migration and differentiation. Toll-like receptors (TLRs)-mediated innate immune response..
2	KIAA1324	Endosome-Lysosome Associated Apoptosis And Autophagy Regulator 1	1.64	2.25e-11	Apoptosis and cell proliferation.
3	HIST1H2BO	H2B Clustered Histone 17	-1.19	9.53e-10	Histone activity.
4	CCL2	C-C Motif Chemokine Ligand 2	-3.84	1.22e-09	Cytokine, chemotactic activity for monocytes and basophils.
5	TMEM255A	Transmembrane Protein 255A	-1.29	1.22e-09	Upstream of or within response to bacterium.
6	CYP2J2	Cytochrome P450 Family 2 Subfamily J Member 2	-1.30	1.73e-09	Monooxygenase. Drug metabolism, synthesis of cholesterol, steroids and other lipids.
7	HIST1H2BJ	H2B Clustered Histone 11	-0.89	1.73e-09	Histone activity.
8	GPR15	G Protein-Coupled Receptor 15	-0.97	1.73e-09	Protein Coding. Probable chemokine receptor. Alternative coreceptor with CD4 for HIV-1 infection.
9	FAM20A	FAM20A Golgi Associated Secretory Pathway Pseudokinase	1.03	1.73e-09	Likely secreted and may function in hematopoiesis.
10	HERC6	HECT AND RLD Domain Containing E3 Ubiquitin Protein Ligand Member 6	-1.67	2.69e-09	Related Class I MHC mediated antigen processing and presentation and Innate Immune System.
11	NRIR	Negative Regulator Of Interferon Response	-1.79	4.73e-09	A functional long non-coding RNA. May be a negative regulator of interferon response.
12	LY6E	Lymphocyte Antigen 6 Family Member E	-1.69	5.45e-09	Regulates T-lymphocytes proliferation, differentiation, and activation.
13	SSTR3	Somatostatin Receptor 3	-0.92	5.45e-09	Neurotransmission, cell proliferation, and endocrine signaling, inhibiting the release of hormones and other secretory proteins.
14	LAX1	Lymphocyte Transmembrane Adaptor 1	-0.57	5.45e-09	B cell activation; negative regulation of MAP kinase activity; and negative regulation of T cell activation.
15	HIST1H2BH	H2B Clustered Histone 9	-0.96	6.79e-09	Histone activity.
16	USP18	Ubiquitin Specific Peptidase 18	-2.46	6.79e-09	Interferon-induced ISG15-specific protease. Regulates T-cell activation, negative regulation of the inflammatory response triggered by type I interferon.
17	AC013461.1		0.89	6.81e-09	No annotation.
18	HIST1H2BI	H2B Clustered Histone 10	-1.03	2.1e-08	Histone activity.
19	HIST1H2BE	H2B Clustered Histone 6	-0.96	2.1e-08	Histone activity.
20	HIST1H3C	H3 Clustered Histone 3	-1.16	2.3e-08	Histone activity.
21	PNPT1	Polyribonucleotide Nucleotidyltransferase 1	-1.09	2.83e-08	RNA-metabolic processes.
22	ISG15	ISG15 Ubiquitin Like Modifier	-2.37	2.89e-08	Chemotactic activity towards neutrophils, cell-to-cell signaling, and antiviral activity.

23	HIST1H2AL	H2A Clustered Histone 16	-1.08	2.89e-08	Histone activity.
24	GALM	Galactose Mutarotase	-0.86	3.19e-08	Galactose metabolism.
25	HIST1H2BF	H2B Clustered Histone 7	-0.70	3.74e-08	Histone activity.
26	RP11-356C4.6		1.88	8.09e-08	No annotation.
27	SLAMF6	SLAM Family Member 6	-0.46	8.5e-08	Immunoglobulin superfamilym expressed on Natural killer (NK), T, and B lymphocytes. NK cell activation.
28	MX1	MX Dynamin Like GTPase 1	-1.94	1.06e-07	Cellular antiviral response. Induced by type I and type II interferons. Antagonizes the replication process of several different RNA and DNA viruses.
29	LINC00487	Long intergenic non-protein coding RNA 487	-2,19	1.06e-07	RNA gene, affiliated with the lncRNA class.
30	JADE2	Jade Family PHD Finger 2	-0.43	1.08e-07	Histone activity.
31	OAS2	2'-5'-Oligoadenylate Synthetase 2	-1.50	1.11e-07	Innate immune response to viral infection.
32	HSPBAP1	HSPB1 Associated Protein 1	0.45	1.11e-07	Binds to one of the small heat shock proteins, specifically hsp27.
33	IGLV3-21	Immunoglobulin Lambda Variable 3-21	-1.48	1.11e-07	Variable domain of immunoglobulin light chains, antigen recognition.
34	LAMP3	Lysosomal Associated Membrane Protein 3	-2.56	1.11e-07	Protein degradation and cell survival. Autophagic process. May also play a role in dendritic cell function and in adaptive immunity.
35	SUMF1	Sulfatase Modifying Factor 1	0.38	1.13e-07	Enzyme that catalyzes the hydrolysis of sulfate esters.
36	PGAP1	Post-GPI Attachment to Proteins Inositol Deacylase 1	-1.24	1.21e-07	Transport of GPI-anchored proteins from the endoplasmic reticulum to the Golgi.
37	ZNF620	Zinc Finger Protein 620	-0.52	1.21e-07	Negative regulation of transcription by RNA polymerase II.
38	PPM1K	Protein Phosphatase Mg2+/Mn2+ Dependent 1K	-0.72	1.21e-07	Cell survival and development. Targeted to the mitochondria,
39	HIST1H3J	H3 Clustered Histone 12	-1.16	1.22e-07	Histone activity.
40	SOBP	Sine Oculis Binding Protein Homolog	-1.53	1.52e-07	Development of the cochlea. Defects in this gene have been linked to intellectual disability.
41	IGHG3	Immunoglobulin Heavy Constant Gamma 3	-1.95	1.52e-07	Constant region of immunoglobulin heavy chains.
42	LTA4H	Leukotriene A4 Hydrolase	0.89	1.71e-07	Synthesis of leukotriene B4, a proinflammatory mediator. Degrading proline-glycine-proline (PGP), a neutrophil chemoattractant.
43	C19orf66	Shiftless Antiviral Inhibitor of Ribosomal Frameshifting	-0.80	1.79e-07	Interferon stimulated gene (ISG) that inhibits viral replication.
44	RP11-439M11.1		-1.60	1.85e-07	No annotation.
45	KIFC1	Kinesin Family Member C1	-0.95	1.85e-07	Microtubule binding activity.
46	SPATS2L	Spermatogenesis Associated Serine Rich 2 Like	-1.83	1.86e-07	Enables RNA binding activity.
47	TRPS1	Transcriptional Repressor GATA Binding 1	0.61	1.86e-07	Transcription factor that represses GATA-regulated genes.

48	HIST1H3G	H3 Clustered Histone 8	-1.19	1.92e-07	Histone activity.
49	ANKRD50	Ankyrin Repeat Domain Containing 50	0.61	1.93e-07	Endocytic recycling.
50	HIST1H2BM	H2B Clustered histone 14	-1.01	1.99e-07	Histone activity.

Gene annotations were obtained from www.genecards.com.
